# Homogeneity of Needleless Electrospun Nanofiber Mats

**DOI:** 10.3390/nano13182507

**Published:** 2023-09-06

**Authors:** Edona Morina, Marius Dotter, Christoph Döpke, Ilda Kola, Tatjana Spahiu, Andrea Ehrmann

**Affiliations:** 1 Department of Textile and Fashion, Polytechnic University of Tirana, 1019 Tirana, Albaniaikola@fim.edu.al (I.K.); tspahiu@fim.edu.al (T.S.); 2 Faculty of Engineering and Mathematics, Bielefeld University of Applied Sciences and Arts, 33619 Bielefeld, Germany

**Keywords:** poly(acrylonitrile) (PAN), needleless electrospinning, atomic force microscopy (AFM), density, Martindale cycles, layer thickness, areal weight

## Abstract

Nanofiber mats can be electrospun by different techniques, usually subdivided into needle-based and needleless. The latter allow for producing large-area nanofiber mats, e.g., with a width of 50 cm and lengths of several meters, if electrospinning proceeds for several hours, depending on the required thickness. Even spinning smaller samples, however, raises the question of homogeneity, especially if defined mechanical properties or a defined thickness is required, e.g., for filtration purposes. Very often, only the inner parts of such electrospun nanofiber mats are used to avoid too high variation of the nanofiber mat thickness. For this study, we used wire-based electrospinning to prepare nanofiber mats with slightly varying spinning parameters. We report investigations of the thickness and mass per unit area, measured on different positions of needleless electrospun nanofiber mats. Martindale abrasion tests on different positions are added as a measure of the mechanical properties. All nanofiber mats show unexpectedly strong variations of thickness, mass per unit area, and porosity, as calculated from the apparent density of the membranes. The thickness especially varied by nearly one order of magnitude within one sample, while the apparent density, as the most uniform parameter, still showed variations by more than a factor of two within one sample. This shows that even for apparently highly homogeneous areas of such nanofiber mats, variations cannot be neglected for all potential applications.

## 1. Introduction

Electrospinning is a common technique to produce nanofiber mats with dimensions between a few square centimeters and several square decimeters for diverse applications, such as energy harvesting, sensing, biotechnology, or filtration [1,2,3,4]. In the first developed needle-based electrospinning process, a polymer solution or melt is pressed through a spinneret inside a strong electric field, so that a so-called Taylor cone is formed at the tip of the capillary from which a jet arises [5]. Being dragged to the counter-electrode, the solvent is evaporated from the polymer solution, so that nanofibers with typical diameters of some ten to some hundred nanometers are placed on a substrate covering the counter-electrode. This needle-based technique is usually applied to prepare smaller areas of such nanofibrous membranes [5]. Larger areas can be produced with the different needleless techniques which have been developed since, e.g., spinning from a roller, a porous tube, a wire, ball or disk [6,7,8]. Amongst the needleless techniques, diverse electrode shapes have been tested to replace the single needle of the needle-based systems, such as wires, cylinders, disks or others [9,10,11].

Amongst the most important parameters of nanofiber mats prepared from a single polymer, such as poly(acrylonitrile) (PAN), the thickness of the nanofibers, the porosity and pore size distribution of the mat, and the macroscopic membrane thickness can be mentioned [12]. It is well known that the electric field can influence these parameters [13,14,15], so that it can be expected that large nanofibrous membranes spun in a needleless process will have other physical properties at the borders, compared to the middle. Nevertheless, these variations along large nanofiber mats are only scarcely reported in the literature [10,16,17,18,19].

In a previous study, we discussed the dependence of the nanofiber diameter measurements on the choice of micrographs [20], which is why this study concentrates on measuring the macroscopic parameters nanofiber mat thickness, mass per unit area, porosity, and abrasion resistance. The nanofiber mat thickness strongly influences not only its mechanical stability, but is also highly important for applications in filtration [21,22,23], biomedicine [24,25,26], and others. The mass per unit area, also called areal weight [27,28,29], as well as the porosity, which can be measured as an apparent density of the nanofibrous membrane [30,31,32], are also important macroscopic parameters for diverse applications of nanofiber mats in the areas of filtration, biotechnology, energy materials, and others.

Our investigations show for the first time quantitatively the variations of these parameters for optically homogenous, needleless electrospun nanofiber mats with different spinning durations and thus allow discussing the potential variation of the aforementioned macroscopic parameters along such nanofibrous membranes.

## 2. Materials and Methods

Spinning solutions were produced from 16% PAN (X-Pan, copolymer with 6% methyl methacrylate, from Dralon, Dormagen, Germany) dissolved in dimethyl sulfoxide (DMSO, min 99.9%, S3 Chemicals, Bad Oeynhausen, Germany) by vigorously stirring at room temperature for 2 h. Electrospinning was performed in the wire-based machine “Nanospider Lab” (Elmarco, Liberec, Czech Republic) on a polypropylene (PP) nonwoven with the spinning parameters presented in Table 1. These parameters were varied to ensure proper electrospinning at the given environmental conditions.

It must be mentioned that the first two parameters, temperature and relative humidity, could not be fully controlled due to working in a non-air-conditioned environment. While the temperature can only be modified by waiting until a desired value is reached (which was not possible in summertime, when this study was performed), the relative humidity is reduced by using dry compressed air. Naturally, this value decreases with increasing temperature.

Next, the voltage was set by increasing it to a value where electrospinning starts and then slowly decreasing it again to avoid the formation of “cotton candy”, i.e., fibrous connections between both electrodes. While we tried to keep this parameter constant during the study, the different environmental conditions led to different necessities regarding the applied voltage. The current is measured, not controlled, and depends especially on voltage and relative humidity.

The substrate speed was maintained at a low value, with the exception of sample 3 where the influence of a static substrate was tested. Finally, the spinning duration was set as long as possible until either the spinning solution was nearly empty, by this reducing the polymer flow, or the formation of “cotton candy” started.

The spinning parameters are thus not equal for all samples, but had to be adopted to the respective situation, as it is common practice in electrospinning.

The surface morphology was investigated by an atomic force microscope (AFM) FlexAFM (Nanosurf, Liestal, Switzerland) with a Tap 190AI-G cantilever in the dynamic mode. Approach parameters were optimized for the respective surfaces (setpoint 55%, P-gain 550, I-gain 1000 and D-gain 0 or 100).

The nanofiber mat thickness was measured with a J-40-T digital thickness gauge for textiles and nonwovens (Wolf-Messtechnik GmbH, Freiberg, Germany) according to ISO 5084 [33]. This standard describes measuring the distance between the base plate on which the sample is placed and a parallel circular presser-foot which exerts a defined pressure on the fabric under examination. An analytical balance VWR LA Classic (VWR International GmbH, Darmstadt, Germany) was used to measure the sample masses. Both measurements were performed on the pure nanofiber mats without substrates.

The apparent density was calculated from the measured mass, thickness and area of the samples, by dividing the mass by the sample volume. The porosity was calculated by the ratio of the apparent density and the literature value of the bulk density of PAN. A Martindale abrasion tester was applied to investigate the abrasion fastness of the samples.

**Figure 1:**
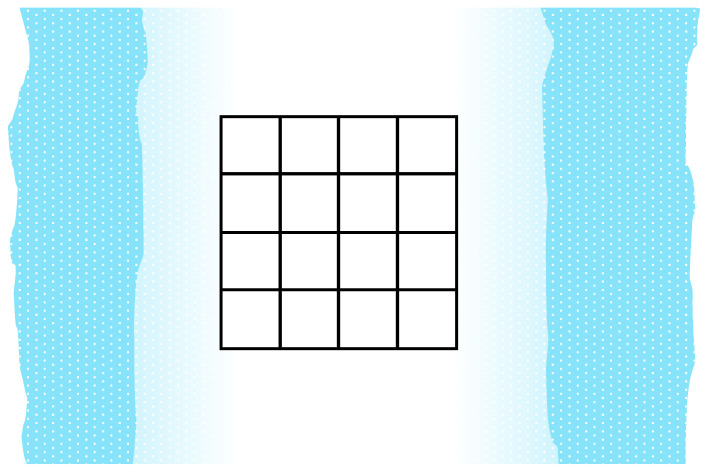
Schematic of the area under investigation (for samples 2–4; for sample 1 an additional row of 4 squares was added). The white nanofiber mat is spun on the light-blue PP nonwoven, where the spinning area is restricted at the top and bottom by the borders of the PP substrate, and at the left and right side by the geometry of the spinning chamber and the substrate speed. The left and right borders are usually thinner due to the decreasing electric field in these areas.

To examine the homogeneity of the samples, the inner (20 cm)^2^ of each specimen were cut into 4 × 4 squares of area (5 cm)^2^ and labeled according to their positions to enable position-dependent comparison. For sample 1, the largest area seemed optically homogeneous, which is why here an area of width 20 cm and height 25 cm was subdivided into 4 × 5 squares. Figure 1 shows a sketch of the investigated area. All 16 (in case of sample 1: 20) marked squares have dimensions of (5 cm)^2^ and are labeled to enable mapping of the measured values to the original positions.

## 3. Results and Discussion

**Figure 2:**
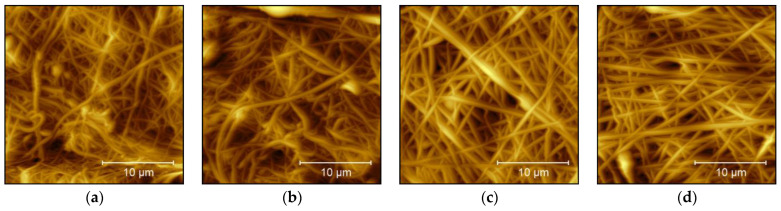
Atomic force microscopy (AFM) images of samples (**a**) 1, (**b**) 2, (**c**) 3, and (**d**) 4.

AFM images were taken on 3–5 arbitrarily chosen positions per sample. Figure 2 depicts typical surface structures found for each sample. All images show fibers with low fiber orientation and similar diameters, as could be expected due to the identical spinning solutions and similar spinning parameters [10]. Some positions on the samples revealed beads (e.g., visible in Figure 2a), which are well known to occur when electrospinning is performed at relatively high humidity, as was the case for the samples under investigation here.

**Figure 3:**
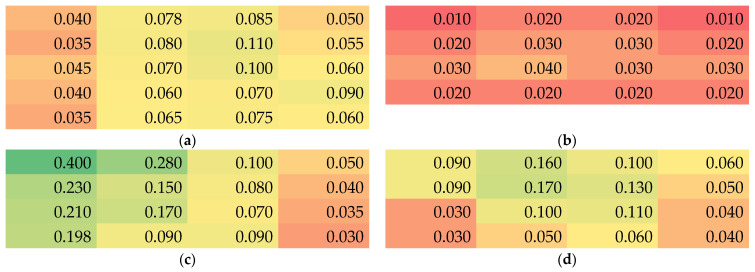
Color-coded thickness values (green for thickest, red for thinnest samples) in mm of the samples 1–4 (**a**–**d**).

Next, the position-dependent thicknesses are depicted in Figure 3. Firstly, it is visible that sample 2 is generally thinner than the others, although the spinning duration was not much shorter than the value of samples 1 and 4. On the other hand, sample 3—whose substrate was not transported during electrospinning and was thus longer spun at the same position—is not significantly thicker than the others. These observations show that even with similar spinning and environmental parameters, the resulting nanofiber mat properties can strongly differ and should always be measured.

Within each nanofiber mat, there are also unexpectedly high variations of the membrane thickness visible. Especially for all nanofiber mats spun with moving substrate (samples 1, 2 and 4), the lengthwise variation (here visible along the rows) is much higher than expected, often showing deviations by a factor of 2 or even higher. Similarly, the variations along the columns are partly very strong, although only an intermediate area of 20–25 cm height was taken into account, as compared to the full membrane height of 50 cm. This again shows the low reproducibility of the nanofiber mat thickness even in the center part of the electrospun membrane.

It must be mentioned that these large deviations, partly by nearly one order of magnitude between the thickest and the thinnest part of the nanofiber mat, are unexpected. Generally, it could be expected that along the left and right border (Figure 1), the nanofiber mat should become thinner since the electric field is weaker further away from the wire electrodes in the middle. However, these borders, where the nanofiber mats were visibly thinner, were not taken into account, but only the middle area which optically seemed to be homogeneous.

One possible explanation could be that the naturally occurring inhomogeneities during spinning the first layers, based on jets from polymer solution starting at arbitrary wire positions and landing on arbitrary positions on the substrate, would not level out during spinning for longer durations, but rather intensify. This would be the case if the dielectric constants of the PP substrate and the PAN nanofibers differed strongly, as has been shown in previous investigations [15]. However, the dielectric constant of PAN (~3 [34]) and of PP (~2 [15]) are quite similar, so that this explanation is not valid. 

Instead, the electric field distribution along the substrate area must be considered. Indeed, Forward and Rutledge calculated the angular-dependent electric field distribution for a wire-based electrospinning system and found a strong angle-dependence [35]. In our electrospinning system with an electrode–substrate distance of 240 mm and the borders of the examined area being 100 mm away from the middle for the static case (Figure 1), resulting in a maximum angle of approx. 23°. For this angle, Forward and Rutledge calculated a reduction in the electric field for an applied voltage of 30 kV from approx. 70 kV/cm to approx. 40 kV/cm, i.e., nearly a reduction by a factor of 2. While the modeled geometry does not fit perfectly, this estimation already shows that larger deviations than originally assumed can be expected here.

Furthermore, it should be mentioned that in case of the moving substrates, there may be an additional time-dependence of the spinning flow, which can shift the maximum thickness away from the middle of the whole electrospun area. Here, however, a difference between left and right border is mostly visible in sample 3 which was spun on a static substrate. A possible explanation for this finding is that the middle of the sample, which was optically determined by the apparently thickest area between the visibly thinner borders, was not perfectly met. Another potential explanation is given by unobserved strands of “cotton candy”, i.e., nanofibrous connections between both electrodes, which may have formed near the beginning of the spinning process. Such “cotton candy” strands can be released from the spinning electrode and completely placed on the substrate, where it would no longer be visible after the full spinning duration. This procedure can take less than the (here) usually 5 min between optical inspections of the spinning process and thus happen without observation.

**Figure 4:**
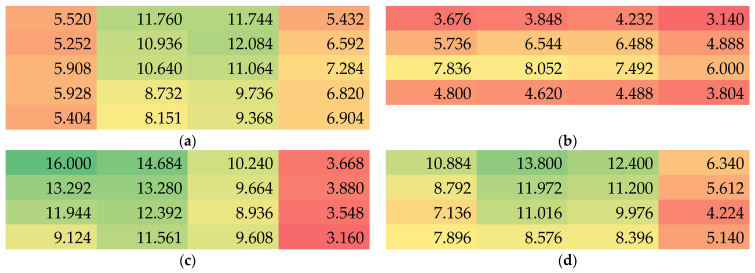
Color-coded values of the mass per unit area in g/m^2^ of samples 1–4 (**a**–**d**).

Besides the thickness, the masses per unit area were measured. The results are depicted in Figure 4. Here, samples 1, 2 and 4 show column-wise relatively consistent values, while the values again differ strongly along the rows. In all samples, the middle parts show higher masses per unit area, indicating that only a small area around the spinning wire position may show approximately homogeneous areal weights.

**Figure 5:**
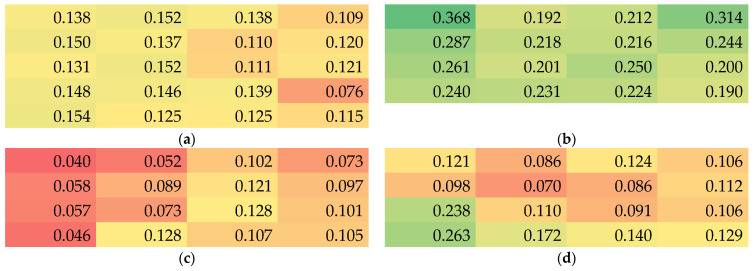
Color-coded values of the apparent density in g/cm^3^ of samples 1–4 (**a**–**d**).

The apparent density, calculated as the measured mass divided by the measured volume of the specimens, is shown in Figure 5. This value is, on the one side, more homogeneous within each specimen than the previous parameters. On the other hand, not only the thinnest mat shows the largest apparent densities, but within each membrane, a tendency is visible that thicker parts show a lower apparent density. This finding may be correlated with the modification of the electric field in the spinning chamber by the dielectric properties of the already spun nanofibers [14]. On the other hand, it is possible that the apparent density is reduced by unobserved strands of “cotton candy” on the substrate or on the first nanofiber layers, as explained above.

**Figure 6:**
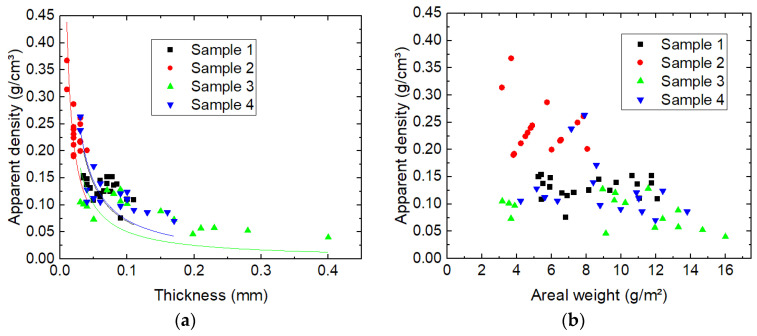
Correlations between the apparent density and (**a**) the membrane thickness, measured data with 1/x fits for all samples in the corresponding colors; (**b**) the mass per unit area (here abbreviated as areal weight).

To investigate whether this correlation is real, Figure 6 depicts the apparent density vs. the thickness and vs. the mass per unit area, respectively. Indeed, Figure 6a shows an approximately antiproportional correlation which can well be fitted by 1/x curves. Figure 6b, however, does not reveal any correlation between apparent density and mass per unit area (areal weight). Nevertheless, all data points can be found in a lower left triangle, i.e., there are no data points with large areal weight and large apparent density at the same time.

**Figure 7:**
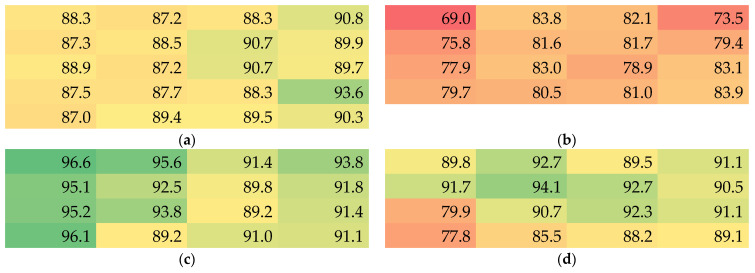
Color-coded values of the porosity in % of samples 1–4 (**a**–**d**).

The density of bulk PAN is 1.184 g/cm^3^, allowing for calculating the porosity of the samples, which is given in Figure 7. While for the thinnest mat (sample 2, Figure 7b), values between approx. 69% and 84% porosity are found, the thicker mats show porosity values of up to more than 96%. It should be mentioned that, as discussed before, a gradient of the porosity can be assumed, with lower values in the first layers of nanofibers and higher porosity in the subsequent layers. Nevertheless, even for the thinnest membranes, high values of more than 2/3 pore volume inside the overall nanofiber mat volume are measured. Using the same simple measurement method via the apparent density, other researchers found porosity values around 63–90% [36,37,38], which is similar to the values reported here.

**Figure 8:**
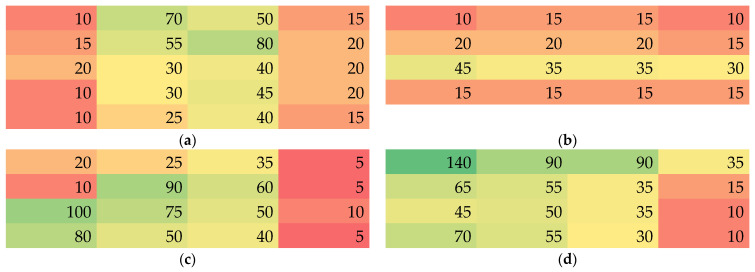
Color-coded values of the number of Martindale abrasion cycles until a hole was visible in samples 1–4 (**a**–**d**).

Finally, Martindale abrasion tests were performed. The values in Figure 8 depict the number of Martindale cycles after which the first hole was visible. It should be mentioned that according to the state of the optically detected damage after a certain number of Martindale cycles, the next optical inspection was performed after 5, 10 or 15 further cycles, since optical examination after each single cycle is technically challenging.

Here again, large differences within a single nanofiber mat are visible. It should be mentioned that values of some ten Martindale abrasion cycles or even more than 100 cycles, as visible for one position in sample 4, are unexpectedly high. Usually, nanofibers spun on different substrates could be expected to be abraded after few abrasion cycles [39,40]. These nanofibrous coatings, however, are usually very thin, e.g., electrospun for only 8–30 min [39,40], as opposed to the nanofiber mats used here. During the Martindale tests it could be observed that the nanofibers were peeled off the membrane layer by layer, so that thicker nanofiber mats with higher areal weight should indeed be able to withstand the Martindale abrasion test longer.

**Figure 9:**
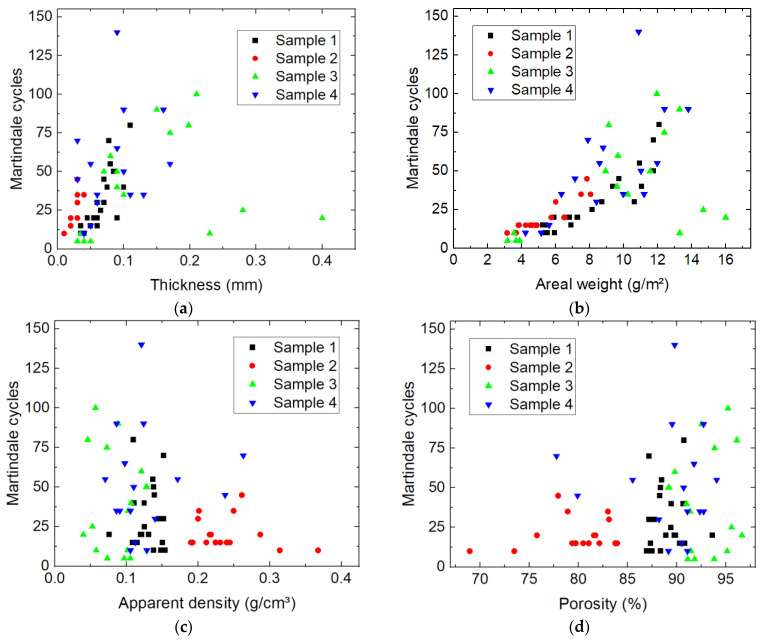
Correlations between the number of Martindale cycles before destroying the sample and (**a**) the membrane thickness; (**b**) the mass per unit area (here abbreviated as areal weight); (**c**) the apparent density; and (**d**) the porosity of the specimens.

Comparing these values with the spatial distributions of thickness, mass per unit area, apparent density and porosity, respectively, suggests a correlation of the number of Martindale cycles with the specimen thickness. For a quantitative investigation of potential correlations, Figure 9 shows the number of Martindale cycles before destroying the sample vs. the aforementioned sample parameters.

As assumed before, a tendency of more Martindale cycles for thicker membranes (Figure 9a), as well as for nanofiber mats with higher mass per unit area (Figure 9b), is visible. The three outliers belong to sample 3 (on the lower right side of Figure 9a,b) may be due to cotton candy-like agglomerations in the sample which can occur during electrospinning, especially if the substrate is not moving at all. Such agglomerations would only increase the thickness in a small area, leading to an unreliable thickness value in Figure 9a, and would make the sample more prone to breaking upon abrasion. This explanation also fits the finding that these three values belong to small apparent densities and high porosities (Figure 9c,d), corresponding to an erroneously high thickness measurement.

While the trend to showing a positive correlation between Martindale cycles and thickness or areal weight, respectively, was expected, no such correlation is visible for the comparison of the Martindale cycles with the apparent density and the porosity, respectively (Figure 9c,d). It can only be stated that abrasion-resistant samples with high numbers of Martindale cycles do not have a high apparent density or low porosity, respectively.

## 4. Conclusions

The homogeneity of electrospun poly(acrylonitrile) (PAN) nanofiber mats was investigated in terms of their thickness, mass per unit area, apparent density, porosity, and number of Martindale cycles necessary to produce a hole in the sample. Correlations were found between the apparent density and the inverse sample thickness, between the number of Martindale cycles and thickness, as well as mass per unit area.

Thickness and areal weight especially varied strongly within each nanofiber mat, although only the optically homogeneous inner parts of the electrospun samples were investigated. These findings show that applications of nanofibrous membranes need clear specifications of suitable parameter ranges, and that neither taking all specimens for a certain investigation from the same sample, nor spinning several samples under similar conditions, will ensure reliable, homogeneous physical parameters of the investigated nanofiber mats.

In the future, tensile tests should be added, as their results are not only based on the parameters examined here, but also on the fiber–fiber friction which partly depends on the evaporation of the solvent before the nanofibers are placed on the substrate. In addition, a systematic evaluation of the effect of the substrate speed, which was only changed from slowly moving to being static here, must be added, since a larger substrate speed may result in leveling out inhomogeneities due to the modifications of the electric field around the wire position.

## Figures and Tables

**Table 1:** Spinning parameters for the samples prepared in this study.

Parameter	Sample 1	Sample 2	Sample 3	Sample 4
Temperature	20.3 °C	22.4 °C	26.0 °C	25.3 °C
Rel. humidity	33%	33%	31%	31%
Voltage	58 kV	60 kV	61 kV	53 kV
Current	0.022 mA	0.032 mA	0.045 kA	0.024 kA
Carriage speed	100 mm/s	100 mm/s	100 mm/s	90 mm/s
Substrate speed	2 mm/min	2 mm/min	0 mm/s	2 mm/min
Nozzle diameter	0.8 mm	0.8 mm	0.8 mm	0.8 mm
Duration	68 min	54 min	45 min	62 min

## Data Availability

All relevant data produced in this study are presented in this paper.
